# Cerebrovascular Management Considerations in Patients on AATs

**DOI:** 10.3390/jcm14103420

**Published:** 2025-05-14

**Authors:** Dylan Ryan, Wuwei Feng, Andy J. Liu

**Affiliations:** Department of Neurology, Duke University School of Medicine, Durham, NC 27704, USA

**Keywords:** dementia, amyloid, stroke, hemorrhage, anticoagulation, left atrial appendage, ARIA-E, ARIA-H

## Abstract

The prevalence of Alzheimer’s Disease (AD) is increasing worldwide, with more emergency providers and neurologists expecting to encounter these patients. The paradigm of management of AD is expected to change given the recent approval of anti-amyloid therapies (AATs). The most concerning complication of these therapies is amyloid-related imaging abnormalities (ARIA), which can lead to an increased risk of cerebrovascular complications. Given a growing population of patients with AD and growing use of AATs, providers must be prepared to manage patients at risk of cerebrovascular disease and those presenting with neurologic deficits. This subpopulation warrants a unique approach given the risk of ischemic stroke and the associated risk of hemorrhage present in the use of AATs. In this narrative review, we present and propose management considerations in the acute stroke setting and patients at risk of cerebrovascular disease, including patients with indications for anticoagulation, to most appropriately manage this special population. Future cross-disciplinary collaboration and use of registry data will be essential to narrow management approaches and develop safety data.

## 1. Introduction

There are approximately 55 million people worldwide diagnosed with dementia [1], which is expected to double by 2040 [1]. The most common cause of dementia is Alzheimer’s Disease (AD), which accounts for approximately 60–70% of cases, with an incidence that markedly increases after the age of 65 [2]. By age 80, about 40% of individuals have amyloid pathology [3]. AD often coexists with vascular disease and related risk factors, which contribute to cognitive decline. One-third of patients presenting with new ischemic stroke are over the age of 80 [4], and many are also diagnosed with dementia. The National Institute on Aging defines dementia as the loss of cognitive function severe enough to interfere with daily life [5]. Patients with AD are at risk of both ischemic and hemorrhagic events [6], complicating stroke management within this population.

The paradigm of management in AD dementia will likely change with the recent development of anti-amyloid therapies (AATs). Aducanumab was the first drug approved in the United States through the accelerated Food and Drug Administration (FDA) pathway in 2021 after showing a reduction in the Clinical Dementia Rating Sum of Boxes (CDR-SB) in the EMERGE trial (difference −0.39; 95% CI, −0.69 to −0.09; *p* = 0.012) [7] in patients with mild cognitive impairment (MCI) and early AD dementia. However, this medication has not received full FDA approval to date. This was subsequently followed by full FDA approval of lecanemab with a reduction in the CDR-SB at 18 months in the CLARITY-AD trial (difference, −0.45; 95% CI, −0.67 to −0.23; *p* < 0.001) [8] in patients with MCI and early AD dementia in 2023. Similar results have been seen with donanemab in patients with MCI and early AD dementia in the Trailblazer-ALZ trial regarding change in CDR-SB score (difference, −0.7; 95% CI, −0.95 to −0.45; *p*  < 0.001) [9], leading to FDA approval for this drug in July 2024. The development of these therapies has led to considerable hope among providers and patients, given the mechanism of action in showing that removal of amyloid beta could lead to slowed progression of their symptoms.

The incidence of acute ischemic stroke is expected to more than double over the next 20 to 30 years, with the majority of the increase being among the elderly (≥75 years old); the number of AIS patients who have dementia will continue to rise proportionately. It is expected that the utilization of AATs will increase dramatically in the coming years. These therapies are also being actively studied in patients with positive AD biomarkers without clinical symptoms, or could even be used off-label, and thus could have even more utility as we learn more about their efficacy within this population. Considering the high impact on mortality and disability related to stroke [10] on a global scale in conjunction with increasing disability related to AD, continuing to preserve or prolong quality of life in these patients is paramount. Stroke providers should expect to encounter more patients being managed with these therapies in the next 1–2 years, which could create further diagnostic, therapeutic, and even ethical challenges regarding stroke intervention in the acute setting.

A significant challenge in patients with AD treated with AATs regarding stroke intervention is amyloid-related imaging abnormalities (ARIA). ARIA-E (edema) refers to magnetic resonance imaging abnormalities representing vasogenic edema secondary to changes in the blood-brain barrier related to these drugs, while ARIA-H (hemorrhage) represents aberrations in signal abnormalities related to microhemorrhages, macrohemorrhages, superficial siderosis, and hemosiderosis that can present with symptomatic intracranial hemorrhage (sICH) [11]. These imaging-related changes are thought to be secondary to the monoclonal antibodies binding accumulated amyloid-beta within the vasculature, leading to amyloid clearance and loss of vessel wall integrity. Moreover, an immunological response to the monoclonal antibody is thought to cause the vasogenic edema and hemorrhages seen on MRI brain imaging [12]. The imaging abnormalities can further be graded (Table 1) [13] and change the patient’s overall management [12]. The incidence of ARIA with the use of each AAT can be found in Table 2. Incidence further increases with the presence of the APOE ε4 allele, with the highest rates in patients homozygous for APOE ε4 [8,9,13] as seen in Table 2. The majority of cases are asymptomatic [8], with rates of symptomatic ARIA-E and ARIA-H being 2.8% and 0.7% with lecanemab [8], and a rate of serious ARIA of 1.6% in patients on donanemab [9].

Given the vascular impact of AATs, there must be differential considerations in the treatment approach of patients in the acute stroke setting and antithrombotic management in patients at risk of stroke. In this review, we aim to outline acute treatment considerations and antithrombotic management options in patients receiving AATs.

## 2. Role of Intravenous Thrombolysis

Intravenous thrombolysis (IVT) with Alteplase has been proven to improve functional outcome in patients presenting within 4.5 h of acute ischemic stroke (AIS) [14,15], which has led to guidelines that recommend IVT in eligible patients presenting within this window [16]. Alzheimer’s Disease and other related dementia (ADRD) are not a contraindication for the use of IVT in eligible patients presenting with acute ischemic stroke; however, this population has been underrepresented in trials related to stroke, considering many have excluded patients older than 80 years old. The European Stroke Organization guidelines have since recommended the utilization of IVT in patients older than 80, though an individual risk and benefit assessment remains standard in the assessment of the use of intervention within this population [17].

The presence of >10 cortical cerebral microbleeds (CMBs) has been shown to be associated with a higher risk of sICH in patients treated with IVT [18,19], which has led to many providers deferring use of IVT in older patients with cognitive impairment. The treatment effect of IVT is also modified in older patients with more severe strokes in those with >10 CMBs, as it is associated with higher mortality [19], signifying that patients with a high pretest probability of >10 CMBs may warrant a different IVT pathway. Though there has been noted to be an increased risk of sICH in patients with >10 cerebral microbleeds (CMBs), the overall rates of sICH with the use of IVT in patients with CMBs have been shown to be as low as 2% [18]. An analysis of the WAKE-UP trial found no reduced treatment effect of IVT in patients with ≥1 CMB, while there were higher rates of sICH [20]. In patients with dementia as compared to those without, observational evidence has suggested similar ability to retain pre-stroke disability with use of IVT, though with increased mortality in patients with dementia [21].

Given the associated incidence of ARIA with use of these AATs, the growing number of patients presenting for the first-ever stroke > 80 years of age and increasing use of acute intervention for acute ischemic stroke, emergency providers and vascular neurologists must be mindful of the safety, efficacy, and approach to patients on these therapeutics presenting within the interventional window. There has been a documented case of multiple intraparenchymal hemorrhages in a 65-year-old APOE ε4 homozygote treated with IVT while on lecanemab [22] who eventually expired due to complications secondary to the multifocal hemorrhages. A second case involving a 72-year-old APOE ε4 heterozygote receiving donanemab who was treated with IVT, who passed from the development of bilateral intracerebral hemorrhage in the setting of severe ARIA-E [23]. These cases have led to significant concerns in the stroke provider community and family/caregivers regarding the use of IVT within this population. Therapeutic use guidelines for lecanemab do not recommend use of IVT within this population [24], though it remains unclear if use of AATs should be a clear contraindication in all patients.

In patients with a risk of cerebrovascular disease, it is important to provide anticipatory guidance to patients to discuss their preferences in the acute stroke setting. Given ARIA can mimic stroke, emergency care providers and vascular neurologists must be aware of patients receiving these therapies when considering management. Markers in the electronic medical record or physical notices of use of AATs could also aid in making sure safe decisions are made within the IVT window. Use of hyperacute MRI could also be considered in these patients to help aid in risk-benefit discussions regarding IVT decisions to attempt to stratify risk weighing against a reduced benefit of thrombolysis with increased time to therapy [19], as traditional non-contrast CT paradigms would be unable to effectively detect evidence of ARIA. Previous modeling has suggested that the justifiable time to obtain an MRI to aid in IVT decision making based on the degree of CMBs is less than 10 min [19], though given the incidence of ARIA in this population, it is possible this paradigm would be different in this special subpopulation. Questions regarding the APOE ε4 genotype and overall risk with the use of IVT within this population should also be considered when faced with these decisions, given their increased risk of ARIA, and therefore knowing the patient’s underlying genotype could aid in predictive value and stratification. In patients with acute stroke on MRI and no evidence of ARIA, there would remain a theoretical risk, but appropriate risk-benefit discussions could be performed with the patient, family, and providers based on their wishes to aid in this management decision.

## 3. Endovascular Mechanical Thrombectomy

In addition, the current practice guideline by the American Heart Association/American Stroke Association (AHA/ASA) is to offer a mechanical thrombectomy (MT) to those patients with an arterial ischemic stroke (AIS) involving a large vessel occlusion (LVO), but only if there is no history of disability, including no Alzheimer’s disease and other related dementia (ADRD) [16]. The guideline was based on several initial pivotal stroke interventional trials demonstrating the safety and efficacy of MT in the setting of AIS. However, due to patient selection bias and inclusion/exclusion criteria, these trials excluded or minimally included patients with ADRD; thus, there is a lack of evidence for the utility of MT in this special but expanding population. A newly published scientific statement [21] by the AHA/ASA strongly supports the need for a randomized clinical trial of efficacy, safety, and cost-effectiveness of MT in this population. The Patient-Centered Outcome Research Institute (PCORI) has funded a research project to evaluate the effectiveness and safety of endovascular intervention in stroke patients with existing disabilities, including dementia [25].

In patients with known cerebral microbleeds, evidence suggests that MT performed without the use of IVT does not increase the risk of sICH [26]. In a study that included 28 patients with probable cerebral amyloid angiopathy (CAA), the MT was found to be beneficial regarding functional outcome at 90 days, with or without concurrent IVT, though worse compared to patients without CAA [27]. Given these findings, the use of CT angiography to assess for large vessel occlusion would be warranted in patients on AATs presenting with stroke symptoms. The DIRECT-MT trial [28], in patients with LVO, directly routing patients to MT was found to be noninferior to bridging with IVT. Given the potential risks in patients eligible for both IVT and MT in this population, deferring IVT in favor of progressing directly to MT could be a preferable strategy to theoretically minimize overall bleeding risk while maximizing the potential benefit of acute therapy.

## 4. ICH Management

Rates of sICH in patients prescribed AATs related to severe ARIA-H are <1% [8,9,13], though this remains an overall concern. ARIA-E and ARIA-H may present simultaneously, given the shared mechanism impacting vascular permeability in patients prescribed AATs [29]. ARIA-H has also been observed to develop in regions previously impacted by ARIA-E [30], with sequential occurrence suggesting the areas of previous edema may be more susceptible to hemorrhage. Observational study of patients on donanemab found lower mean arterial pressure and use of antihypertensives medications were predictive of reduced rates of ARIA-E [31], potentially suggesting a role in reducing rates of hemorrhage in this population with strict blood pressure control. In patients presenting with focal neurologic deficits, receiving AATs and imaging to rule out sICH is warranted. In those with observed ICH, there is no current evidence to deviate from ICH management guidelines [32]. In patients with cerebral microbleeds, minimally invasive evacuation does not appear to carry increased risk of harm [33,34] and thus should be considered in patients prescribed AATs. In patients presenting with ICH while using AAts, MRI is warranted to aid in diagnosing severe ARIA-H, which can also coincide with ARIA-E [35], given management considerations for severe ARIA.

## 5. Management of ARIA

Clinical symptoms concerning for ARIA, including focal neurologic deficits, seizure, new onset headaches, and encephalopathy, should prompt MRI evaluation for ARIA. In patients with symptomatic cases meeting criteria for moderate or severe ARIA (Table 1), treatment with AATs should be held [24], and monthly monitoring with MRI should be performed. With resolution of changes or clinical stability, clinicians can reconsider re-initiation of therapy, discussing overall risk and benefits. Rates of recurrent ARIA after re-initiation of therapy can be as high as 25% in patients [36].

Patients with severe ARIA noted on MRI should be further evaluated in a hospital setting. As previously noted, patients with macrohemorrhages should be treated according to clinical guidelines [32] in the appropriate setting. In patients with CAA-related inflammation (CAA-ri), which can present similarly to severe ARIA, treatment has involved high-dose corticosteroid pulses over 3–5 days followed by a prolonged oral taper over up to six months [37], and in some cases, progression to use of cyclophosphamide. Patients with CAA-ri have responded well to immunosuppression with positive responses in up to 75% of patients [37,38].

Similar to those with CAA-ri, patients with severe ARIA should be treated with pulse methylprednisolone over 3–5 days, followed by a prolonged oral steroid taper [24,39]. Patients should be monitored for complications of therapy, including hypertension, hyperglycemia, and neuropsychiatric symptoms. ARIA typically resolves over a course of months without further complications [40]. In this group of patients, anti-amyloid therapy should not be re-initiated given the overall risk.

## 6. Antiplatelet Management

Patients with AD often have concurrent vascular risk factors and underlying indications for antiplatelet medication. In older patients, there is no indication for the use of antiplatelet agents for the primary prevention of cerebrovascular or cardiovascular disease [41,42]. In those with documented cardiovascular disease, antiplatelet agents provide a clear benefit and thus are commonly prescribed in older patients with concomitant dementia. Antiplatelet agents do increase the risk of bleeding, and thus, questions regarding the use of these agents have been raised in patients prescribed AATs.

In patients with CAA, antiplatelet therapy has been associated with the increased risk of sICH [43,44,45], further raising concerns regarding use in patients prescribed AATs. Given that ARIA can lead to changes similar to those in patients with CAA, there existed concerns about continuing antiplatelet therapy in this population. In safety results regarding lecanemab, antiplatelet monotherapy appeared to be well tolerated with no increased risk of ARIA [40]. There is limited data regarding the use of dual antiplatelet therapy (DAPT) in patients on AATs, though long-term use of DAPT is known to significantly increase the risk of ICH [46]. DAPT, most commonly with aspirin and clopidogrel, has been found to reduce the risk of stroke in those with high-risk transient ischemic attack (TIA) and minor stroke [47,48], though the overall risk profile may be very different in patients using AATs. Standard monotherapy doses appear reasonable to continue in this population with continued standard monitoring for those on anti-amyloid therapy.

## 7. Anticoagulation Management

In patients with indications for anticoagulation, there is a known reduction in the incidence of stroke and cerebrovascular disease. One of the most common reasons for patients to receive anticoagulation is atrial fibrillation (AF). Advancing age is the most important non-modifiable risk factor for the development of AD, though this is also true for AF [49]. It is currently estimated that the population of adults older than 65 years old will double by 2040 [50], which will further increase the disease burden, particularly in the United States, Europe, and Asia, related to AF. Given the increasing incidence and prevalence of both AD dementia and AF after the age of 65, it will not be uncommon to see patients who carry both diagnoses, and given the increasing age of the global population, it can be anticipated that more patients could be impacted by both pathologies.

The use of anticoagulation, as compared to antiplatelet monotherapy, has been shown to significantly reduce the risk of systemic embolism and stroke [51]. Direct oral anticoagulants (DOACs) have also been found to reduce the risk of bleeding compared to vitamin K antagonists [52,53,54,55] and thus have been a mainstay in therapy. Bleeding risk with apixaban, a commonly used DOAC, has been found to be similar to that of aspirin monotherapy [51,56], leading to further use of these agents.

In patients with cerebral microbleeds and AF, therapeutic anticoagulation is noted further to increase the risk of ICH [57]. In patients receiving concomitant lecanemab and anticoagulation, there was an ICH rate of 2.4% in the phase 3 study and 2.9%, including the open-label extension, compared to 0.1% in the placebo arm [40]. There was a reported death of a patient in his late 80s due to an ICH in the CLARITY-AD trial [8] who was being managed with apixaban while on lecanemab [58], though his death was later reported to be felt to be unrelated to lecanemab use. These findings have led to significant concerns about the utilization of anticoagulation in patients potentially eligible for the use of AATs at risk of cerebrovascular disease.

In patients at high risk of bleeding, there has been real-world use of low-dose DOACs. Low-dose DOACs are commonly used in clinical practice in patients meeting specific criteria; however, in real-world clinical practice, close to 50% of patients receiving low-dose apixaban do not meet these criteria [59]. An analysis of 1722 patients on low-dose rivaroxaban and 3833 on low-dose apixaban found no difference in stroke in the low-dose rivaroxaban group as compared to the standard dose, though risk of stroke in low-dose apixaban as compared to standard dose was higher (HR, 1.95; 95% CI, 1.38–2.76) [60] without reduction in bleeding. Given the potential risk of ICH in patients receiving AATs, more data would be needed prior to advocating for the use of anticoagulation at any dose. Current appropriate use recommendations for lecanemab exclude the use of oral anticoagulants [24]. The behavioral neurologists at Duke exclude patients on DOAC who are otherwise eligible for lecanemab.

One possible consideration regarding management of patients with comorbid AF and MCI or early AD dementia could be left atrial appendage (LAA) closure, given questions regarding the safety of oral anticoagulants within this subgroup (Table 3). The majority of studies on the efficacy of this approach in the management of AF and stroke risk reduction have been performed using the WATCHMAN device (Boston Scientific, Marlborough, MA, USA) [61]. The PROTECT-AF trial compared the WATCHMAN to warfarin in patients with nonvalvular AF and found LAA closure with the WATCHMAN was noninferior regarding risk of stroke, systemic embolism, and cardiovascular death (HR, 0.61; 95% CI, 0.38–0.97; *p* = 0.04) [62]. The PREVAIL trial [63] compared patients with AF and a CHADS2 score of ≥2 to LAA closure with the WATCHMAN versus warfarin and found LAA closure was noninferior to warfarin in terms of stroke and systemic embolism. An analysis in the two trials found LAA closure to be associated with a lower risk of hemorrhagic stroke, cardiovascular death, and nonprocedural bleeding as compared to warfarin [64], making it an attractive alternative to anticoagulation in patients at risk of hemorrhage. This approach has previously been utilized in patients with CAA and has been shown to be feasible and safe [65], though more studies would be needed to assess overall outcomes. These patients often do require a short course of anticoagulation preceding and after the procedure, which does have to be weighed given this class of medications with the patient. It is possible that this approach would reduce overall risks related to AF on brain health while also allowing patients with AD pathology to have access to disease-modifying therapy. This approach has been successfully used in a 77-year-old patient with newly diagnosed AF on lecanemab without vascular complications [66].

The LARIAT device has also been FDA-approved for soft tissue closure, but it has been used for LAA closure. The LARIAT is an epicardial closure device that, unlike the WATCHMAN, may not require a course of anticoagulation, given that no foreign body remains on the epicardial surface. A study on 21 patients using the device found 100% successful LAA closure with no observed strokes within a mean of 352 ± 143 days of follow-up [67]. A subsequent study on 89 patients found 96% of patients underwent successful LAA closure with two late strokes thought to be non-embolic in nature [68]. A 27-patient study found 88% LAA closure at four months on transesophageal echocardiography, but it did have one periprocedural stroke related to thrombus formation on the transseptal sheath [69]. A prospective, single-center study on 139 patients found the thromboembolism rate was 0.6% with a calculated thromboembolism risk reduction of 81% [70], suggesting the safety and efficacy of this approach. Given that this approach could lead to a lower burden of need for short-term anticoagulation, it could be an attractive option to allow patients with comorbid AF and AD to reduce cardiovascular risk while also gaining access to disease-modifying therapy for AD.

The AtriClip is an epicardial clipping device used to occlude the LAA that has been shown to have a near 100% success rate on imaging of occluding the LAA [71,72]. In a study of 291 AtriClip device deployments in patients with a mean CHA2DS2-VASc of 3.1, there was an ischemic stroke rate in patients who discontinued oral anticoagulation of 0.5/1000 patient-years as compared to an expected rate of 4/1000 patient-years [73] without evidence of procedural complications. Given the epicardial nature, there is no requirement for the use of postprocedural anticoagulation, which could make this a viable approach within this patient population.

LAA closure and epicardial clipping do not come without risks. Periprocedural risks include the risk of bleeding, infection, pericardial effusion, and anesthesia-related complications, the latter of which may be higher risk in those with dementia. Endocardial devices carry the risk of device embolization and device-related thrombi, both of which are rare [64] but can occur. Device leak can also occur, which can lead to the need for repeat imaging and monitoring. Epicardial clipping carries the risk of injury to surrounding structures, such as the pulmonary artery or phrenic nerve, and clip migration, though these appear to be rare [72]. These approaches also possess less robust evidence of benefit as compared to therapeutic anticoagulation, though use in practice and study populations is increasing.

**Table 3:** Summary of studies assessing stroke and embolism risk with the use of left atrial appendage closure devices.

Study	Device	Study Type	Implantation Success	Results
Reddy et al. (PROTECT-AF) [74]	WATCHMAN	Prospective, randomized, multi-center clinical trial Warfarin control arm	88%	Composite stroke/systemic embolism/cardiac death: HR 0.71 (95% CI, 0.44–1.30)
Holmes et al. (PREVAIL) [63]	WATCHMAN	Prospective, randomized, multi-center clinical trial Warfarin control arm	95%	Composite stroke/systemic embolism/cardiac death: RR 1.07 (95% CI, 0.57–1.89)
Litwinowicz et al. [70]	LARIAT	Prospective, observational, single-center	96.4%	Thromboembolism rate: 0.6% (Estimated risk reduction 81%) Severe bleeding rate: 0.8% (Estimated risk reduction 78%)
Caliskan et al. [73]	AtriClip	Prospective, observation, multi-center registry	100%	Ischemic stroke rate w/o anticoagulation: 0.5/1000 patient years (Estimated risk reduction 87.5%)

## 8. Conclusions and Future Directions

Given that clinicians can expect to encounter more patients being managed with AATs, protocols should be developed to aid in management decisions in the emergency setting and clinical setting related to cerebrovascular disease. At this time, vascular neurologists and emergency physicians must approach these patients with great caution when considering IVT and/or MT. A registry to carefully document both short-term and long-term outcomes of these patients will provide valuable guidance to the stroke community. Reverse translation research using preclinical rodent models may be considered to sort out these scientific issues and further inform clinical decisions in this population. In patients with focal, disabling neurologic symptoms, it is possible that hyperacute MRI could be used to aid in diagnostic certainty and in assessing overall risks of use of IVT within this specific population, though this would need further study. Utilization of MRI remains important in diagnosing cases of severe ARIA to aid in symptomatic therapy using corticosteroids, as well as in diagnosing new evidence of cerebrovascular disease. Reports of outcomes in patients with LVO undergoing MT will also be beneficial in assessing outcomes in this population, as well as reports based on MT technique, as aspiration versus the use of stent retrievers could have differing complications. We look forward to solutions in this special but expanding population in the near future, backed by rigorous studies as the use of these anti-amyloid therapies becomes more routine.

Registry data collecting outcomes in patients on AATs with comorbid AF could help behavioral neurologists, vascular neurologists, and cardiologists understand the risk and benefit and make better treatment decisions in the future regarding anticoagulation. Prospective study on the utility of LAA closure within this unique population could help assess the safety and efficacy of this approach and could lead to increased eligibility for patients diagnosed with these pathologies. Studies assessing how LAA closure compares to the utility of anticoagulation on cognitive outcomes, given the link between these pathologies, could also provide further insight into the value of our treatment decisions in this population. Pre-clinical models assessing the risks of anticoagulation with concomitant use of AATs could also provide needed insights on the safety of DOACs in patients on anti-amyloid therapies that could aid in future clinical studies and clinical decision making for providers.

Collaboration of behavioral neurologists, vascular neurologists, emergency physicians, and interventionalists will aid in developing optimal treatment strategies for patients presenting with neurologic deficits and those at risk of neurologic deficits. Further long-term evidence regarding the safety and impact of AATs will also be beneficial in further adjusting protocols to ensure safe but effective management strategies in this special population of patients.

## Figures and Tables

**Table 1:** Definition of radiographic severity of ARIA-E and ARIA-H.

ARIA Subtype	Radiographic Severity		
	Mild	Moderate	Severe
ARIA-E	FLAIR hyperintensity confined to a sulcus, cortex, or subcortical white matter confined to one location < 5 cm	FLAIR hyperintensity of a single lesion measuring 5–10 cm or more than one site < 1 cm	FLAIR hyperintensity > 10 cm with significant subcortical white matter and/or sulcal involvement with one or more sites of involvement
ARIA-H microhemorrhage	≤4 new incident microhemorrhage	5–9 new incident microhemorrhages	≥10 new incident microhemorrhages
ARIA-H superficial siderosis	1 focal area of superficial siderosis	2 focal areas of superficial siderosis	>2 focal areas of superficial siderosis

**Table 2:** Incidence of ARIA-E, ARIA-H, and any ARIA in clinical trials of aducanumab, lecanemab, and donanemab in patients with early AD.

Drug	Number of Subjects	Incidence ARIA-E—APOE ε4 Noncarrier	Incidence ARIA-E—APOE ε4 Carrier	Incidence ARIA-H—APOE ε4 Noncarrier	Incidence ARIA-H—APOE ε4 Carrier
Aducanumab (10 mg/kg) [13]	1029	20.3%	43.0%	12.4%	22.7%
Lecanemab (10 mg/kg) [8]	859	5.4%	15.8%	11.9%	19.7%
Donanemab (700 mg for 3 doses then 1400 mg) [9]	853	15.7%	27.1%	19.7% (not reported separately)	
